# Bridging the Gap: Combining Genomics and Transcriptomics Approaches to Understand *Stylosanthes scabra*, an Orphan Legume from the Brazilian Caatinga

**DOI:** 10.3390/plants12183246

**Published:** 2023-09-13

**Authors:** José Ribamar Costa Ferreira-Neto, Manassés Daniel da Silva, Eliseu Binneck, Natoniel Franklin de Melo, Rahisa Helena da Silva, Ana Luiza Trajano Mangueira de Melo, Valesca Pandolfi, Fernanda de Oliveira Bustamante, Ana Christina Brasileiro-Vidal, Ana Maria Benko-Iseppon

**Affiliations:** 1 Laboratório de Genética e Biotecnologia Vegetal, Center of Biosciences, Genetics Department, Federal University of Pernambuco, Av. Prof. Moraes Rego, 1235, Recife 50670-901, PE, Brazil; rahisa.silva@ufpe.br (R.H.d.S.); analuiza.melo@ufpe.br (A.L.T.M.d.M.); valesca.pandolfi@ufpe.br (V.P.); fernanda.bustamante@uemg.br (F.d.O.B.); ana.vidal@ufpe.br (A.C.B.-V.); 2 Laboratório de Genética Molecular, Center of Biosciences, Genetics Department, Federal University of Pernambuco, Av. Prof. Moraes Rego, 1235, Recife 50670-901, PE, Brazil; manasses.dsilva@ufpe.br; 3 Brazilian Agricultural Research Corporation’s—EMBRAPA Soybean, Rodovia Carlos João Strass—Distrito de Warta, Londrina 86001-970, PR, Brazil; eliseu.binneck@embrapa.br; 4 Brazilian Agricultural Research Corporation’s—EMBRAPA Semiárido, Rodovia BR-428, Km 152, s/n-Zona Rural, Petrolina 56302-970, PE, Brazil; natoniel.melo@embrapa.br

**Keywords:** nuclear genome, aquaporins, mobile elements, R-genes, PRR-genes, drought

## Abstract

*Stylosanthes scabra* is a scientifically orphaned legume found in the Brazilian Caatinga biome (a semi-arid environment). This work utilized omics approaches to investigate some ecophysiological aspects of stress tolerance/resistance in *S. scabra*, study its genomic landscape, and predict potential metabolic pathways. Considering its high-confidence conceptual proteome, 1694 (~2.6%) proteins were associated with resistance proteins, some of which were found in soybean QTL regions that confer resistance to Asian soybean rust. *S. scabra* was also found to be a potential source of terpenes, as biosynthetic gene clusters associated with terpene biosynthesis were identified in its genome. The analysis revealed that mobile elements comprised approximately 59% of the sequenced genome. In the remaining 41% of the sections, some of the 22,681 protein-coding gene families were categorized into two informational groups: those that were specific to *S. scabra* and those that expanded significantly compared to their immediate ancestor. Biological process enrichment analyses indicated that these gene families play fundamental roles in the adaptation of *S. scabra* to extreme environments. Additionally, phylogenomic analysis indicated a close evolutionary relationship between the genera *Stylosanthes* and *Arachis*. Finally, this study found a high number (57) of aquaporin-encoding loci in the *S. scabra* genome. RNA-Seq and qPCR data suggested that the PIP subfamily may play a key role in the species’ adaptation to water deficit conditions. Overall, these results provide valuable insights into *S. scabra* biology and a wealth of gene/transcript information for future legume omics studies.

## 1. Introduction

The Caatinga is exclusively a Brazilian biome. This phytogeographic domain covers most of the semi-arid region of the country [1]. Its flora is considered one of the most fascinating due to plant morphological and molecular adaptations to a semi-arid and hostile environment. Water availability is scarce, and rainfall regimes fluctuate significantly [2]. This scenario directly interferes with the distribution and survival of different life forms [3]. Scientific projections for the Caatinga indicate an increase in average temperatures of up to 4.5 °C and a reduction in rainfall of up to 50% by the end of the 21st century [4].

Some legumes in the mentioned area are important crops, with a strong impact on the lives of locals. Commonly, these plants have a low availability of molecular information and are thus scientifically considered orphans. However, these rustic species have adapted to withstand environments with severe growth conditions. They have evolutionarily developed morphological, physiological, biochemical, and molecular mechanisms to survive drought, high temperatures, and high salinity, among other extreme edaphoclimatic factors common to the Caatinga phytogeographical domain. It is imperative to identify the genetic properties of resilient crops, with the aim of developing new strategies to obtain commercial crops adapted to current climate changes.

Among the legumes found in the Caatinga, there are species of the *Stylosanthes* (subfamily: Papilionoideae) genus. Their ability to restore fertility and improve soil physical properties is notable because of their association with rhizobia (nitrogen-fixing bacteria). Additionally, species of the aforementioned genus are forage (i.e., they provide permanent vegetation cover and serve as food for animals [5]). These findings indicate the economic importance of this plant group.

In Brazil, there are approximately 30 perennial species of *Stylosanthes* [6]. *S. scabra*, commonly found in the Caatinga region, stands out for its drought tolerance [7] and its ability to grow in moderately acidic soils with low fertility [8]. This species is often found in regions of high salinity. *S. scabra* has substantial osmotic adjustment capacity and maintains tissue turgor even with low water potential in the environment. Additionally, there are scientific reports indicating moderate resistance of some *S. scabra* accessions to pathogenic agents, such as the fungus *Colletotrichum gloeosporioides*, which causes anthracnose [9,10]. This disease is the most important and widespread for the genus *Stylosanthes* [10] and also affects a range of economically important crops, such as sorghum, wheat, beans, and soybeans [11]. Due to the presented properties (a stress-tolerant forage legume), *S. scabra* was introduced in the regions of Australia and Hawaii [12].

Despite these robust ecophysiological traits, *S. scabra* has minimal omics resources to decipher molecularly. Our research group recently analyzed physiological/biochemical parameters and RNA-Seq libraries of this species under water deficit conditions [13]. To date, this is the only study to present transcriptomic data for *S. scabra*. For the genus Stylosanthes, there are reports of only four more studies [14,15,16,17] addressing high-throughput gene expression studies, all on *Stylosanthes guianensis*.

The genome of *S. scabra*, in turn, has been studied primarily using cytogenetic strategies (through methods such as fluorescence in situ hybridization (FISH) and genomic in situ hybridization (GISH) [18]) or molecular markers (ISSR markers, for example [19]). Marques et al. [18] also focused on sequencing and comparing plastomes (chloroplast genome, specifically) and rDNA (ribosomal DNA) sequences between different species of the genus Stylosanthes (including *S. scabra*), performing an evolutionary analysis. For the referred clade, to date, there has been no high-throughput sequencing data for the nuclear genome. Furthermore, significant gene families with profound physiological effects need to be explored in *S. scabra*, taking into account its structural genomics and transcriptomics under stressful conditions.

Hence, the current study aimed to bridge the information gap reported above by using high-throughput sequencing, assembly, and investigation of the *S. scabra* nuclear genome. Additionally, genomics and transcriptomics of the aquaporin gene family have been studied. Plant aquaporins are membrane proteins that play critical roles regulating water movement across plant cell membranes [20,21]. They are essential for plant growth, development, and adaptation to various environmental stressors such as drought, high salinity, and extreme temperatures [20,21].

*S. scabra* represents a valuable source of genes, gene families, and molecular mechanisms that need to be studied and further explored. The flora of the Caatinga yields wealth that drives its development. This genetic wealth may, therefore, be a starting point for the generation of knowledge and contribute to the sustainability of agriculture through biotechnology and all related fields.

## 2. Material and Methods

### 2.1. Plant Material and Genomic DNA Extraction

The plants used for genomic DNA extraction were derived from the work of Ferreira-Neto et al. [13]. The seeds of *S. scabra* “85/UNEB” accession (ID: 85/UNEB) were formally obtained from the “Active Germplasm Bank of the Universidade do Estado da Bahia” (UNEB), Brazil. Prof. Dr. Natoniel Franklin de Melo (EMBRAPA Semiárido) kindly carried out the species/accession identification. No voucher specimens were created. The cultivation process is briefly described as follows.

The *S. scabra* plants were propagated using stem cuttings at Embrapa Semiárido (Petrolina, Pernambuco, Brazil). Stem cuttings (10 cm in length) were subjected to application of 1000 ppm of indolebutyric acid (IBA) dissolved in mineral talc and transplanted into 15 × 8 cm plastic bags (three seedlings/pot) containing ultisol and vermiculite (3:1) as substrate. The cuttings were kept in a climate-controlled greenhouse (50% shade) under irrigation (twice a day).

After rooting, the plants were transferred to plastic pots (10 L)—containing substrate comprising sand-ultisol-vermiculite—and maintained in a greenhouse under controlled conditions of temperature (25 ± 2°C), humidity (60 ± 5%), and a 12 h/day natural light photoperiod (photosynthetic photon flux density (PPFD) = 1.5 × 10^3^ μmoles m^−2^s^−1^ per 12 h/day).

After six months of cultivation, genomic DNA was extracted from the young leaves using a modified CTAB method [22], and the concentration and purity of the obtained biomolecule were assessed using a Qubit Fluorometer (Thermo Fisher Scientific, Waltham, MA, USA) and NanoDrop^®^ 2000 (Thermo Fisher Scientific), respectively.

### 2.2. Sample Sequencing and Genome Size Estimation by Flow Cytometry

Four genomic libraries were constructed using an Illumina Nextera™ DNA Flex Library Preparation Kit, following the manufacturer’s recommendations. Genomic DNA was subjected to 151-bp paired-end Illumina sequencing using an Illumina NovaSeq 6000 sequencer.

The S. scabra genome size was calculated using a modified version of the methodology proposed by Dolezel et al. [23]. To liberate the nuclei, fresh leaves were chopped in 1 mL of GPB (general purpose buffer [24]) together with an internal reference standard (Zea mays L.; 2C = 5.33 pg). After the tissues were cut, the nuclei were stained with 60 µL of 1 mg/mL propidium iodide and passed through a 30 µm nylon mesh filter. Using Partec CyFlow Space, at least 5000 nuclei from three different runs over several days were examined. FlowMax v.2.7 was also used to create flow cytometry histograms, and the average ratio between the fluorescence intensity peak values of the samples and the reference standard was used to determine the 2C value for the studied species.

### 2.3. S. scabra De Novo Genome Assembly and BUSCO Analysis

In total, 2 × 626.3 million paired-end sequences were generated. Before assembly, the sequencing data were processed in paired-end mode using Trimmomatic version 0.39 [25] for quality trimming and primers/adapters clipping, with the following parameters: phred33, ILLUMINACLIP: NexteraPE-PE.fa:2:30:10, LEADING: 3, TRAILING: 3, SLIDINGWINDOW: 4:15, MINLEN: 91.

Quality checks of the reads were performed before and after trimming using FastQC version 0.11.9 (http://www.bioinformatics.babraham.ac.uk/projects/fastqc; accessed on 10 January 2023) and MultiQC version 1.10.1 [26]. After trimming, 1.15 billion paired reads (with average lengths of 149 bp) and 38.4 million single-end reads (with average lengths of 138 bp) were used for de novo assembly. For this step, Velvet version 1.2.10 [27] was used with VelvetOptimiser version 2.2.6 (https://github.com/Slugger70/VelvetOptimiser; accessed on 10 January /2023) to test the hash lengths (k-mers) 91, 99, and 107.

The VelvetOptimiser automatically optimized the Velvet’s primary parameters “exp_cov” and “cov_cutoff”. The assembled genome was evaluated using QUAST-LG version 5.0.2 [28] with the *Arachis hypogaea* genome (NCBI RefSeq GCF_003086295.2) as a reference.

The gene space completeness assessment of the genome assembly was carried out using gVolante [29] and the ortholog search pipeline “BUSCO” v.5 (ortholog set: Fabales). gVolante report scores were based not only on the coverage of reference genes but also on sequence lengths (fox example, N50 scaffold length), which allows for quality control in multiple aspects.

### 2.4. Gene Prediction, Functional Annotations and General Gene Features

RNA-seq raw data from *S. scabra* [13], summing a set of 116 million read pairs after trimming (TrimmomaticPE with parameters ILLUMINACLIP: TruSeq3-PE.fa:2:30:10, SLIDINGWINDOW: 4:5, LEADING: 5, TRAILING: 5, MINLEN: 25), were applied to produce transcript evidence using PASA version 2.4.1 [30]. The protein-coding gene models were predicted using GeneMark-ET version 4.68 [31] and AUGUSTUS version 3.3.3 [32], using PASA transcript evidence to improve training.

The consensus structures for gene annotations were computed using EVidenceModeler [33] and updated using PASA. Transfer RNA (tRNA) genes were predicted using tRNAscan-SE version 2.0 [34]. Putative functions of protein-coding genes were predicted by pattern matching with the Pfam [35], UniProtKB/Swiss-Prot (https://www.expasy.org/resources/uniprotkb-swiss-prot; accessed on 25 January 2023), eggNOG [36], CAZy [37], MEROPS [38], BUSCO/eudicotyledons_odb10 [39], and InterPro [40] databases. 

The *S. scabra* gene general features were analyzed via Genestats script (https://gist.github.com/darencard/fcb32168c243b92734e85c5f8b59a1c3; accessed on 25 January 2023). The following parameters were scrutinized: (1) transcript sequence length, (2) number of exons, (3) total exon sequence length, (4) number of introns, (5) total intron sequence length, (6) number of CDS chunks, (7) total CDS sequence length, (8) number of 5” UTR sequences, (9) total 5′ UTR sequence length, (10) number of 3′ UTR sequences, and (11) total 3′ UTR sequence length.

In the present work, high-confidence genes were considered those that were predicted by the abovementioned pipeline and that encode proteins with available domain information and/or EC number and/or GO (gene ontology) terms and/or EggNog OGs (ortholog groups).

### 2.5. Transposable Elements and Other Repetitive Sequences’ Mining and Annotation

An *S. scabra* repeat sequence database was built using the principles of de novo and homology predictions by combining RepeatModeler2 [41] and RepeatMasker (https://www.repeatmasker.org/; accessed on 10 February 2023) software, respectively. The first tool is a de novo transposable element (TE) family identification and modeling package. Three de novo repeat sequence mining algorithms (RECON, RepeatScout, and LTR Retriever) are at the core of RepeatModeler. They use complementary computational methods to determine repeat element boundaries and family relationships from sequence data. This action generated a high-quality library of *S. scabra* TE families.

After this step, the output from the referenced software was merged with the interspersed or low-complexity DNA sequences, in addition to TE sequences, of *Arabidopsis thaliana*, from the Repbase (RepBaseRepeatMaskerEdition-20181026.tar.gz) database, to form the final repeat sequence library. This final entity was then used by the RepeatMasker tool to predict and quantify the repeat sequences from the *S. scabra* genome.

### 2.6. Gene Family Identification and Respective Expansion/Contraction Analysis

To identify *S. scabra* gene families, conceptual proteome data (primary proteins only) were downloaded (Phytozome database) from 11 different plant species (besides *S. scabra*): eight Fabaceae (*Vigna unuiculata*, *Phaseolus vulgaris, Glycine max, Cicer aeritnum*, *Cajanus cajan*, *Lotus japonicus*, *Arachys hypogeae*, and *Trifolium pratense*); one Euphorbiaceae (*Manihot esculenta*); one Salicaceae (*Populus trichocarpa*); and one Brassicaceae (*Arabidopsis thaliana*). All data were subjected to similarity analysis using the DIAMOND tool [42], e-value < 1 e^−5^, and the resulting data were grouped into orthogroups (gene families) using Orthofinder software v2.5.5 [43].

To predict the expansion and contraction of the *S. scabra* gene families and to infer species-specific loci gains or losses, the orthogroups by Orthofinder were analyzed using the CAFE5 software v5 [44], with default parameters. A dated species tree was downloaded from the TimeTree database [45] and used as a guide tree (for divergence time estimation between *S. scabra* and *Arabidopsis thaliana* (the outgroup used)). The species tree used in the present study was inferred using STAG (https://github.com/davidemms/STAG; accessed on 1 March 2023) and rooted using STRIDE (https://github.com/davidemms/STRIDE; accessed on 1 March 2023), both of which are contained in the Orthofinder tool. The CAFE5 lambda parameter (birth-death rate) was estimated using gene families in which no more than 100 genes were derived from a single genome. Gene families with a significant rate of expansion or contraction were determined using a threshold conditional *p*-value (*p* < 0.05).

### 2.7. Gene Ontology Enrichment Analysis

GO term enrichment (biological processes, specifically) analysis was performed using PlantRegMap [46] with the singular enrichment analysis method and summarized/visualized using REVIGO [47]. The *S. scabra* conceptual proteome, annotated (BLASTp; e-value < e^−10^) against *Arachis hypogaea*, *Arachis ipaensis*, and *Arachis duranensis* sequences deposited in the Uniprot database (https://www.uniprot.org/; accessed on 10 March 2023), was used as the background.

### 2.8. “R” and “PRR” Gene Mining and Identification

The identification of “R” (resistance) and “PRR” (pattern recognition receptors) genes was performed on a genomic scale using the RRGPredictor tool [48]. To this end, the high-confidence conceptual proteome from *S. scabra* genome was first analyzed by InterProScan5 version 5.51-85.0 (docker image available at https://hub.docker.com/r/interpro/interproscan; accessed on 10 March 2023), using the default databases (COILS, Gene3D, HAMAP, MOBIDB, PANTHER, Pfam, PIRSF, PRINTS, ProDom, PROSITE, SFLD, SMART, SUPERFAMILY, and TIGRFAM). The output format was a tab-separated value (TSV) file containing the identified protein domains.

Subsequently, the RRGPredictor pipeline was employed. It used the following two scripts: The first, “RRG_DomainDetect”, started with the aforementioned *TSV* file and filtered the domains of interest (CC, NBS, LRR, TIR, RPW8, STK, RLK, PTO-Like, GNK2, and MLO) into independent output files. The second, “ClassRRG”, employed two processes. Initially, all output files generated after running the first script were compared among them, selecting sequence IDs if they intersected in the mentioned files. Sequences were then compared and classified. Finally, separate files for each of the R and PRR gene classes (based on the domain combination) were generated with non-duplicated sequences.

RRGPredictor, based on text mining and set theory, is used to identify “PRR” and “R” genes and classify them into 13 categories: CN, CNL, MLO, N, NL, RLK, RLK-GNK2, RLP, RPW8NL, T, TN, TNL, and UNKNOWN. The “UNKNOWN” class consists of proteins with an LRR domain and a transmembrane region, with or without any other domain not included in the other canonical classes.

### 2.9. In Silico Anchoring of S. scabra “R” and “PRR” Genes in Soybean QTL Regions Associated with Resistance to the Phakopsora pachyrhizi

Because *S. scabra* is moderately resistant to some fungi [9,10], the study of its defense proteins has become relevant. Aiming to add biotechnological potential to the *S. scabra* “R” and “PRR” gene set, their proteins were contrasted against proteins anchored in soybean QTL (quantitative trait loci) regions (Wm82.a2.v1; https://soybase.org/; accessed on 15 March 2023) associated with resistance to the fungus *Phakopsora pachyrhizi*, which causes Asian soybean rust. It is the only fungus for which this type of information is available in the soybean genome.

Data mining was performed in the SoyBase database (https://soybase.org/; accessed on 15 March 2023), specifically in the “List of GWAS QTL” section (https://www.soybase.org/GWAS/list.php; accessed on 15 March 2023). The webpage contains information on the QTL regions characterized in the soybean reference genome (Wm82.a2.v1). Genomic sequences from the two analyzed soybean QTL regions (Asian Soybean Rust 1-g1 and Asian Soybean Rust 1-g2) were retrieved by the “Download track data across region Gm0X:XX..XX” option and subsequent processing of the obtained GFF3 file. This file contained, among other information, gene model IDs (Glyma.XXgXXXXXX.X format) of existing loci at 497 Kb up- and downstream of the SNP (single nucleotide polymorphism) markers associated with the respective analyzed QTLs. Gene model IDs were retrieved using a custom-made Python script. Subsequently, IDs were used to download the respective soybean protein sequences. After this step, similarity (BLASTp; cut-off e-value < e^−10^) and orthology (OrthoFinder pipeline [43]) analyses were performed between the sequences under study.

The physical scrutinized distance of 497 kb (up- and downstream of the SNP markers associated with the respective analyzed QTLs) for loci mining was customized in the present work. For the soybean genome, the referenced measure is associated with an average genetic measure of ~1 cm [49]. One centimorgan is equivalent to a 1% chance that a marker on one chromosome will separate from a second marker on the same chromosome owing to crossing over during a single generation [50]. The coding loci of the recovered soybean proteins are, therefore, colocalized with the SNP markers anchored in the analyzed QTL regions. The physical positions of the mentioned markers were observed using the gBrowser of the SoyBase platform.

### 2.10. Aquaporins Mining and Identification in S. scabra Genome and Transcriptome

To search for potential members of the aquaporin gene family in the *S. scabra* genome (SscAQPs), its high-confidence conceptual proteome was used. A FASTA-format file containing the mentioned protein sequences was scrutinized by the HMMER tool (https://github.com/EddyRivasLab/hmmer; accessed on 20 March 2023) to search for Pfam PF00230 (HMM for major intrinsic proteins (MIPs) domain superfamily) matching. Retrieved sequences with two NPA motifs, six transmembrane domains, and five loops were considered complete aquaporins.

The complete SscAQPs were aligned with AQPs from *Arachis duranensis* [51], *Arachis ipaensis* [51], and *Arachis hypogea* [52] using ClustalW software v2.1 [53]. A phenetic tree was constructed using the Molecular Evolutionary Genetics Analysis (MEGA) tool 7.0 [54] with the maximum likelihood method and 1000 bootstrap resamplings. The SscAQPs were categorized by sequence phenetic analyses.

The analyzed *S. scabra* transcriptome (RNA-Seq libraries) was derived from the assembly and global analysis from Ferreira-Neto et al. [13]. These authors studied how the mentioned plant responds to 24 h of water deficit at the molecular level. Differentially expressed transcripts were those that showed −1 > Log_2_FC > 1, *p*-value < 0.05, and FDR < 0.05. The same steps for mining and classifying SscAQPs at the genomic level were also implemented in the transcriptome.

### 2.11. Identification and Annotation of Specialized Metabolite Biosynthetic Gene Clusters

The plantiSMASH web server [55] was used for mining the specialized metabolite biosynthetic gene clusters in the *S. scabra* genome. The scaffold-level genome in FASTA format and its respective GFF3 file were provided as inputs. The default parameters were adopted for the analysis.

### 2.12. qPCR: Setup, cDNA Synthesis, Efficiency, and Relative Expression Analyses

These analyses were performed according to the MIQE guidelines [56]. The SscAQPs with upregulation indicated in RNA-Seq libraries for 24 h water deficit treatment were selected for qPCR investigation. Three biological and three technical replicates were used to guarantee the statistical reliability of the process. qPCR was performed in 96-well plates at LineGene 9660 (Bioer) using the SYBR-Green detection method.

Aliquots of the same total RNA samples used for RNA-Seq libraries sequencing were employed in this step. Possible genomic DNA (gDNA) contamination, RNA quantity and quality screening, cDNA synthesis protocol, qPCR setup, PCR cycling, amplification efficiency assay, primer pairs design, used reference genes, and melting curves analysis (File S1) were performed according to Ferreira-Neto et al. [13].

The Rest2009 software (standard mode) was used to calculate the relative expression of SscAQPs. Such analysis is based on paired comparisons (of target transcript and reference genes under stress conditions and controls) using randomization and bootstrapping—using the Pair-wise Fixed Reallocation Randomization Test© [57]. Hypothesis testing (*p* < 0.05) was used to determine whether differences in the expression of target transcripts under control and treated conditions were significant.

## 3. Results

### 3.1. S. scabra Genome Assembly: General Data

**Figure 1:**
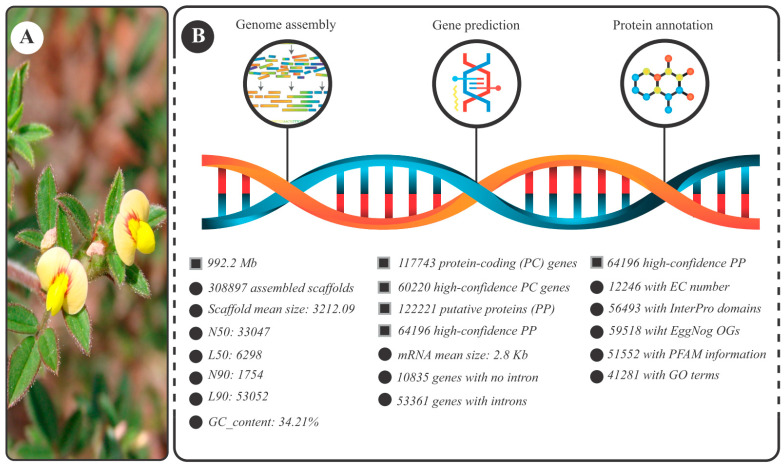
General information about the present study. (**A**) *Stylosanthes scabra* flowering specimen. (**B**) Metrics of the resulting genomic assembly divided into three categories: genome assembly, gene prediction, and protein annotation. Legend: GO terms (gene ontology terms); OGs (orthologous groups); PFAM (protein family); EC (Enzyme Commission); items associated with squares represent topics; items associated with spheres represent subtopics of the topic immediately above.

The *Stylosanthes scabra* (specimen shown in Figure 1A) genome size, estimated by flow cytometry, was ~1.2 Gb. The resulting assembly involved 1252.6 mega-reads, corresponding to 189.14 Gbp. This amount of data resulted in approximately 157-fold genomic coverage. A total of 308,897 scaffolds were obtained (other metrics are presented in Figure 1B). They anchored 992 Mb (Figure 1B), corresponding to approximately 83% of the estimated genome size.

Regarding the protein-coding genes, 117,743 candidate loci were identified (Figure 1B). Of these, 60,220 were considered high-confidence (gene characterization data available in Supplementary Table S1) encoding non-hypothetical proteins, that is, proteins with information on the domain and/or EC number and/or GO terms and/or EggNog OGs terms. These loci encoded 64,196 distinct proteins (Figure 1B).

Regarding the completeness of the obtained genome, BUSCO analysis (File S2) indicated that 92.7% of the single-copy orthologs (core genes) found in the Fabales clade were identified with their complete structure. Recovered genes are classified as “complete” when the lengths are within two mean standard deviations of length of the BUSCO group. The abovementioned BUSCO value raises to 94.4% when considering also the “core genes” found in a fragmented state.

### 3.2. Landscape of the Stylosanthes Scabra Genome Composition

**Figure 2:**
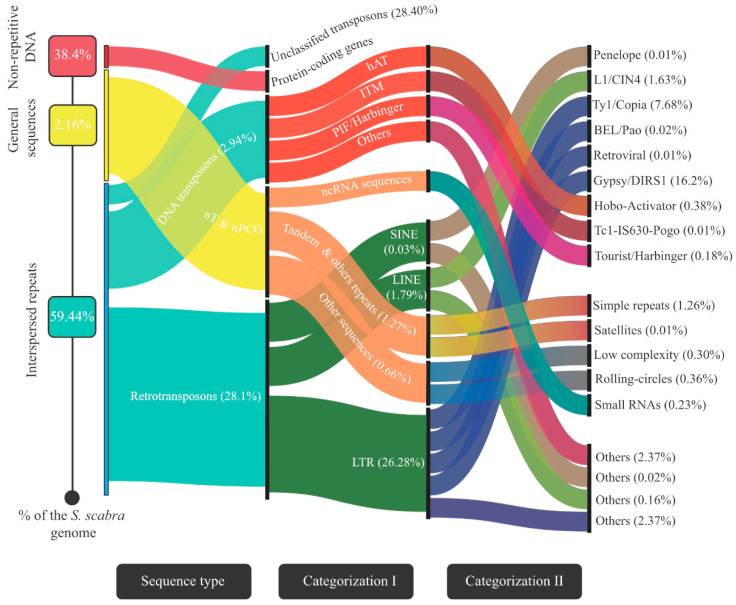
Sankey diagram showing* the different sequence types (interspersed repeats, general sequences, and non-repetitive DNA) found in the *S. scabra* genome, the percentage they occupy in that entity, and their categorization into pertinent sub-items. Legend: * The width of items highlighted in the first and second columns is proportional to the number of sub-items they have. This strategy improves the aesthetics of data presentation due to the wide range between the maximum and minimum values presented. The amounts are presented as follows: the sum of the percentage of child items furthest to the right is equal to the percentage of the parent item immediately preceding it. “nT & nPCG” (non-transposon and non-protein coding gene sequences).

Similar to other eukaryotic genomes, the *S. scabra* genome is predominantly composed of repetitive sequences. These represented 61.6% (i.e., 611,268,409 bp) of the sequenced nucleotides. Figure 2 shows the categorization of the repetitions into groups and their associated subgroups.

Most repetitive sequences are *interspersed* (i.e., they are dispersed throughout the genome). The main representatives of this category were transposition elements, which covered 59.44% of the sequenced genome (Figure 2). They were subdivided into “retrotransposons” (28.1%), “DNA transposons” (2.94%), and “unclassified transposons” (28.4%) based on the adopted annotation pipeline (Figure 2). The most abundant category in the first group was “LTR_Gypsy/DIRS1” (corresponding to 16.2% of the sequenced genome) (Figure 2); for the “DNA transposons” group, the most abundant category was “hAT_hobo-Activator” (representing 0.38% of the sequenced genome) (Figure 2). Regarding the “non-transposon & non-protein coding genes” (nT&nPCG) sequences, “single repeats” (1.26% of the sequenced genome) were the majority (Figure 2). Finally, the 117,743 protein-coding genes (high-confidence or not) mentioned in the previous item were contained in a region that was equivalent to 38.4% (“non-repetitive DNA”; Figure 2) of the analyzed genome.

### 3.3. Identification of S. scabra Immune Receptors and Anchoring Analysis in Soybean QTLs Associated with Resistance to Asian Soybean Rust

**Figure 3:**
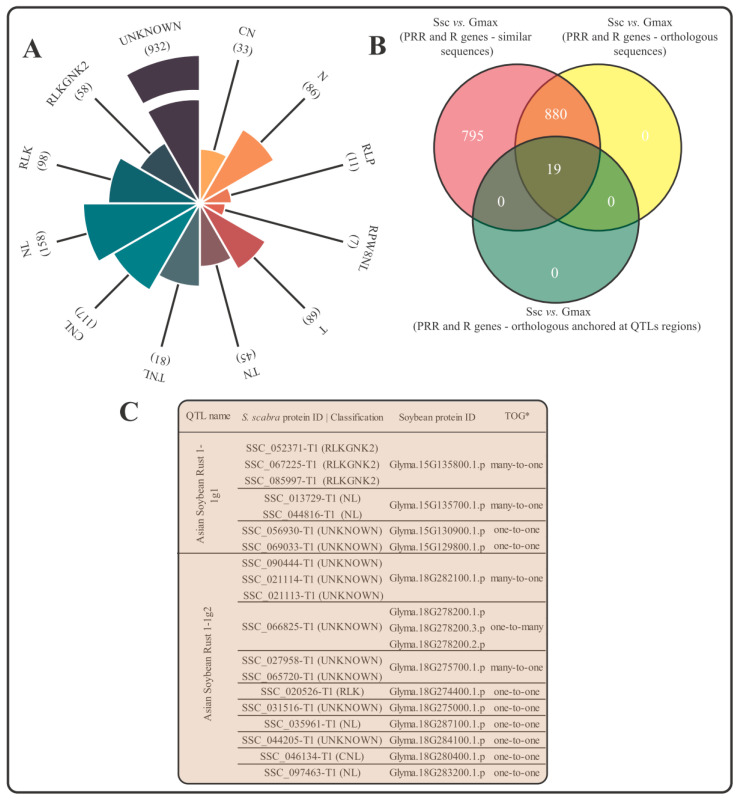
“R” and “PRR” genes mining. (**A**) Classification of “R” and “PRR” genes in the *Stylosanthes scabra* genome. (**B**) Quantitative of *S. scabra* “R” and “PRR” genes that presented orthology with soybean gene loci anchored or not to QTL regions associated with resistance to the fungus *Phakopsora pachyrhizi*. (**C**) Nominal identification of orthologous pairs of *S. scabra* and soybean anchored in the mentioned QTLs and indication of the type of relationship between the pairs. * Types of orthologous groupings (TOG). Legend: Ssc (*Stylosanthes scabra*); Gmax (*Glycine max*).

Due to the already reported moderate resistance of *S. scabra* to the fungus *Colletotrichum gloeosporioides*, mining and identification of “R” (“resistance”) and “PRR” (“pattern recognition receptors”) genes in the legume genome was carried out. These proteins are important plant immunoreceptors that are active for the detection of invading pathogens. Out of 64,196 proteins in its putative high-confidence proteome, 1694 (~2.6%) encoded such protein groups, namely (Figure 3A): “PRR” (98 RLK, 58 RLKGNK2, and 11 RLP), “R” (33 CN, 117 CNL, 86 N, 158 NL, 7 RPW8NL, 68 T, 45 TN, 81 TNL), and “UNKNOWN” class (932). “UNKNOWN”, as mentioned in the Material and Methods section, are proteins with an LRR domain and a transmembrane region with (or without) any other domain not included in the canonical classes defined as “PRR” and “R”.

Analyses by OrthoFinder revealed that, out of 1694 “R” and “PRR” proteins from *S. scabra*, 898 showed orthological relationships with soybean proteins (Figure 3B). To add biological value to these loci, we also scrutinized their possible anchoring in regions of two soybean QTLs associated with resistance to the fungus *Phakopsora pachyrhizi*, which causes Asian soybean rust. To this end, we recovered 208 soybean proteins encoded along the “Asian Soybean Rust 1-1g1” QTL (anchored on the Gm15 chromosome) and 160 proteins encoded along the “Asian Soybean Rust 1-1g2” QTL (anchored on the Gm18 chromosome). In silico, we found 19 orthologs between *S. scabra* and soybean that were co-located in the investigated QTLs. Of the annotated groups (i.e., excluding “UNKNOWN” class), “NL” (4) and “RLKGNK2” (3) emerged as the most abundant categories (Figure 3C). Approximately half of the analyzed loci presented a “one-to-one” orthology relationship in relation to soybean; the other half presented mainly “many-to-one” relationships (Figure 3C).

### 3.4. Biosynthetic Gene Clusters Profile of the S. scabra Genome

As legumes are a remarkable source of specialized metabolites with relevant physiological and ecological functions, and biotechnological use, “biosynthetic gene clusters” (or BGCs) were searched in the genome under study. Despite the fragmented nature of the obtained information (308,897 scaffolds; Figure 1B), the N50 of the resulting assembly (33,047 nt; Figure 1B) provided substantial “raw material” for mining this genomic configuration.

A total of 39 BGCs were identified, ranging in length from 13.6 to 145.25 Kb, and the number of unique enzymes (i.e., functionally distinct enzyme subclasses) ranged from three to six (Supplementary Table S2). Of the BGCs with the “signature” (or “core”) enzyme associated with the synthesis of the backbone of compounds with already known functional groups, those associated with the synthesis of saccharides (13) and terpenes (10) were most prominently represented (Supplementary Table S2). Additionally, a group of 11 putative BGCs also stood out (Supplementary Table S2). These are called so because their “signature” enzymes synthesize compounds whose final chemical class is not properly known.

### 3.5. Gene Families Mining and Analysis of Their Evolutionary Dynamics

The 694,952 primary proteins scrutinized, constituting the 12 analyzed proteomes, formed 38,938 orthogroups (i.e., different gene families). We identified 22,681 distinct gene families encoded in the *S. scabra* genome. Of this number, 4032 did not find representatives in the other evaluated species; they are, therefore, specific to *S. scabra* in relation to the set of analyzed organisms. Such gene families encode 55,081 distinct proteins (approximately 45% of the entire conceptual proteome (high-confidence or not) of the species). An enrichment analysis of this gene group revealed that this group was involved in several biological processes, especially (Supplementary Figure S1) those associated with the cell wall dynamics (“cell wall modification”, “cell wall organization”, “plant-type cell wall modification”, and “lignin metabolic process”), processes associated with terpenoid metabolism (“terpenoid metabolic process” and “diterpenoid biosynthetic process”), and processes associated with development (“regulation developmental process”, “regulation of growth”, and “regulation of leaf development”).

**Figure 4:**
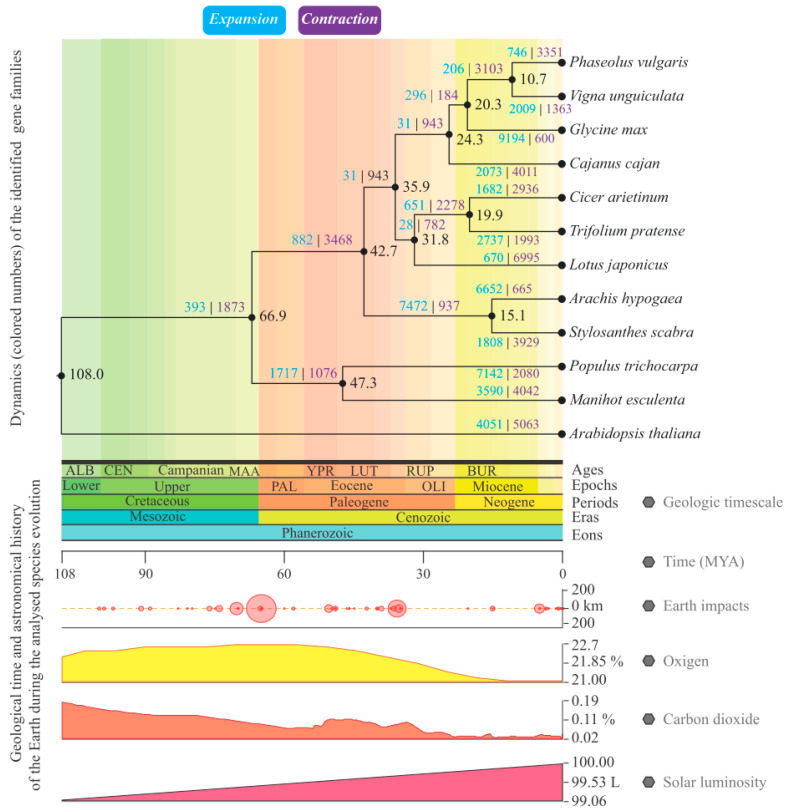
Phylogenetic tree of the 12 analyzed organisms, presenting the expansion/contraction dynamics of their gene families. Legend: Number of expanded (light blue) and contracted (purple) gene families in relation to the last common ancestor of each clade or group of the scrutinized species. At the bottom of the figure, for informative purposes only, contextualization is provided concerning geological and astronomical events that transpired on the planet during the analyzed 108 million years. This timeframe encompasses the divergence between species within the genus Stylosanthes and *A. thaliana* (utilized as an outgroup).

In another context, the dynamics of expansion/contraction of the gene families found in all the scrutinized species was evaluated. Focusing on *S. scabra*, there were 1808 expanded and 3909 contracted gene families (Figure 4). On the other hand, the phylogenetic group most closely related to the species in question, *A. hypogaea,* showed an expansion of 6652 and contraction of 665 gene families (Figure 4).

**Figure 5:**
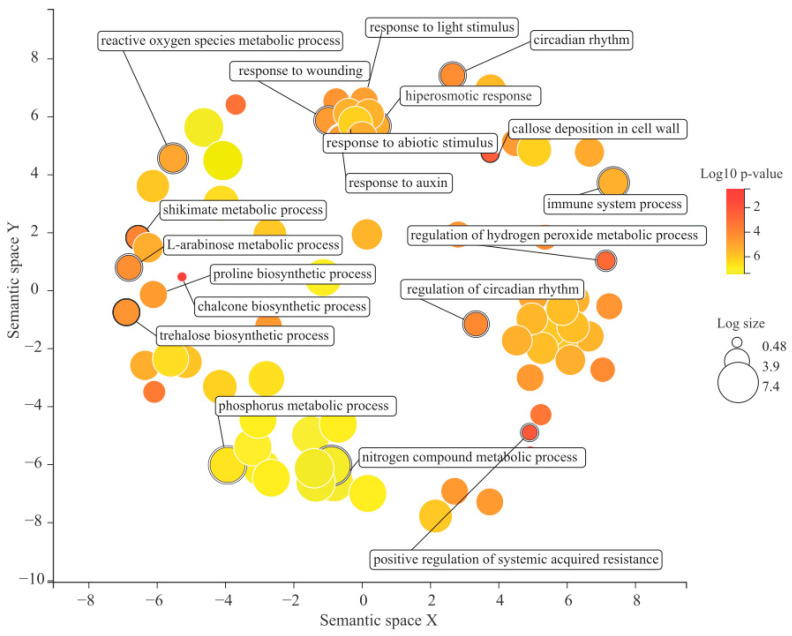
Some of the biological processes enriched for the set of *S. scabra* gene families with significant expansion (*p* < 0.05) in relation to their last common ancestor with *A. hypogaea*. Legend: Bubble color indicates the *p*-value (legend in the upper right-hand corner); “Log size” indicates the frequency of the GO term in the background data used (bubbles of more general terms are larger).

Additional analyses of the *S. scabra* expanded group revealed that 674 gene families showed a significant expansion rate (*p* < 0.05) in relation to the amount of their last common ancestor with *A. hypogaea*. Among the enriched “GO” terms for biological processes carried out by the proteins of that group, the following stands out (Figure 5): those associated with the biosynthesis of secondary compounds or amino acids (example: “trehalose biosynthetic process” and “proline biosynthetic process”), those associated with the reactive oxygen species metabolism (“reactive oxygen species metabolic process” and “regulation of hydrogen peroxide metabolic process”), and those related to the immune system (“immune system process” and “positive regulation of systemic acquired resistance”), among others.

Finally, in the context of comparative phylogenomics, 587 (~87%) of the gene families of *S. scabra* with significant expansion were also significantly expanded (*p* < 0.05) in *A. hypogaea*, which presented significant expansion to 1237 orthogroups.

### 3.6. Aquaporin Gene Family: Genome-Wide Identification and Transcriptomics under Water Deficit

Aquaporins are closely associated with plant responses to stresses causing dehydration [58], which are common in the Caatinga regions. These proteins are involved in the transport of water and small solutes. Due to this importance, this gene family was searched in the genome under study. In addition, the expression of their representatives was evaluated in RNA-Seq libraries from a water deficit assay (24 h irrigation suppression treatment) involving the same accession of *S. scabra*, whose genome was sequenced.

Initially, 79 potential SscAQPs (*Stylosanthes scabra* aquaporins) were identified in the *S. scabra* genome. Subsequent structural characterization (i.e., analysis of the complete presence of NPA motifs, transmembrane domains, and loops; Supplementary Figure S2) of these candidates, however, resulted in the elimination of 22 SscAQPs, leaving 57 canonical SscAQPs associated with 57 different loci (Supplementary Figure S2).

**Figure 6:**
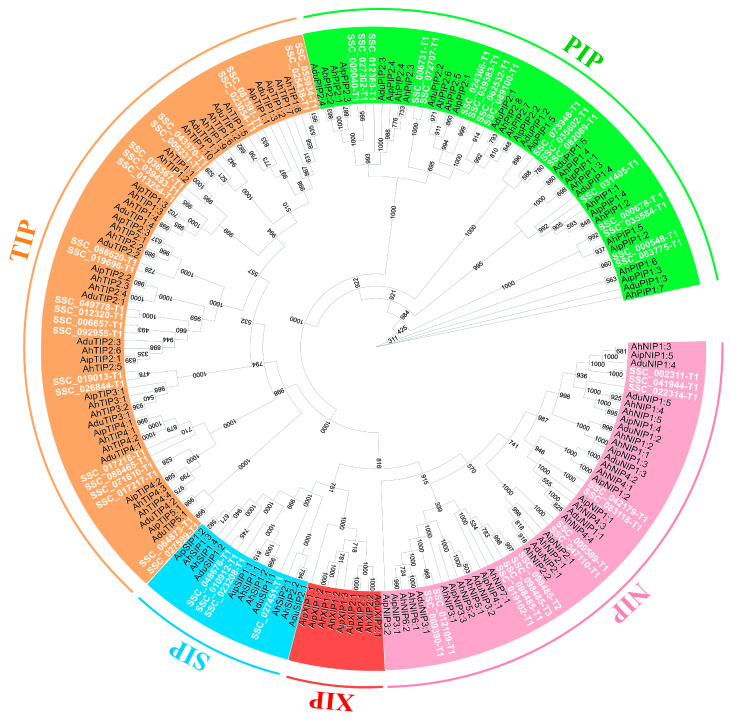
Phenetic tree resulting from alignment of aquaporins from *S. scabra* (Ssc; elements highlighted in white), *A. ipaensis* (Aip), *A. hypogaea* (Ah), and *A. duranensis* (Adu). Legend: Tonoplast intrinsic proteins (TIPs); plasma membrane intrinsic proteins (PIPs); nodulin 26-like intrinsic proteins (NIPs); small basic intrinsic proteins (SIPs); and uncharacterized intrinsic proteins (XIPs). The number in the ramifications represents bootstrap values based on 1000 resamplings.

The phenetic analysis of the canonical SscAQP set, together with AQPs from phylogenetically close organisms such as *A. ipaensis*, *A. hypogaea*, and *A. duranensis*, formed five major groups, four of which have SscAQPs as members (Figure 6). The most abundant group of *S. scabra* was tonoplast intrinsic proteins (TIPs; 23 SscAQPs) (Figure 6). This group was followed by, respectively, plasma membrane intrinsic proteins (PIPs; 17 SscAQPs), nodulin 26-like intrinsic proteins (NIPs; 13 SscAQPs), and small basic intrinsic proteins (SIPs; four SscAQPs) (Figure 6). XIP homologs were not present in the genome.

The comparison of gene characteristics of the different SscAQP families (Supplementary Table S3) revealed that, within each group, the number and length of exons varied little. In contrast, the length of introns oscillated highly, with a variance at least five times greater than that of exons (Supplementary Table S3). The mean number of introns per SscAQP was approximately 2.7 (Supplementary Table S3). With regard to subcellular localization, all SscAQPs were localized in the plasma membrane (Supplementary Table S3).

The functions of aquaporins are delimited by some amino acid residues, mainly those that make up NPA motifs and ar/R selectivity filters. These functional residues are shown in Supplementary Figure S2 and Supplementary Table S3. NPA motifs were conserved in all analyzed groups (Supplementary Figure S2 and Supplementary Table S3). With regard to air/R filters, there was variation both within and between the groups (Supplementary Figure S2 and Supplementary Table S3). The only exception was related to the PIP group, where all representatives presented the amino acids “F”, “H”, “T”, and “R” for “H2”, “H5”, “LE1”, and “LE2”, respectively (Supplementary Figure S2 and Supplementary Table S3).

**Figure 7:**
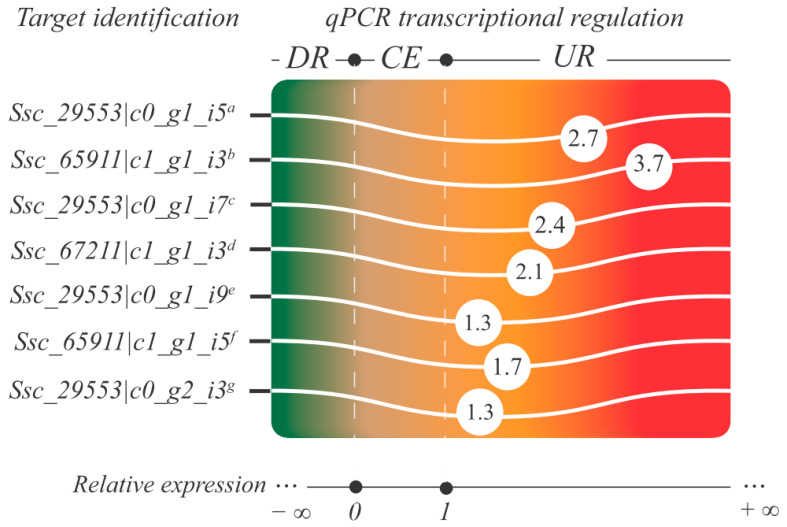
Color gradient graph showing transcriptional regulation of aquaporins analyzed by qPCR. Legend: The IDs represent identifiers in the transcriptome of *S. scabra* produced in response to water deficit (Ferreira-Neto et al. [13]), the respective coding loci in the analyzed genome are presented as overwritten letters (^a^ Ssc_012360, ^b^ Ssc_035602, ^c^ Ssc_023725, ^d^ Ssc_031405, ^e^ Ssc_062532, ^f^ Ssc_026593, ^g^ Ssc_062532), and the numbers inside the circles represent the relative expression values (REST software v1); CE (constitutive expression); DR (downregulation); UR (upregulation).

Regarding transcriptomics, 14 SsAQPs (associated with 11 loci) were upregulated in the root tissue of *S. scabra* subjected to a 24 h water deficit. They are representative of the PIP (nine SsAQPs) and TIP (five SsAQPs) groups (Supplementary Table S4). To validate the RNA-Seq data by qPCR, primer pairs were designed for nine SsAQPs (all PIPs). Of these, seven were functional in preliminary tests (cDNA amplification) and showed efficiency within the adopted cut-off (90–110%) (Supplementary Table S5), being referred for relative expression tests by qPCR. Based on this strategy, the upregulation of all scrutinized PIPs was confirmed (Figure 7; File S3).

## 4. Discussion

### 4.1. Assembly Data and Genomic Composition

This study introduced the first nuclear genome assembly of a species of the genus *Stylosanthes*. Despite the availability of transcriptomic studies [13,14,15,16,17], only one initiative has addressed the genome (in that case, from chloroplast [18]) of representatives of the mentioned genus.

The resultant assembly successfully recuperated approximately 83% (992 Mb out of 1.2 Gb) of the size predicted for the *S. scabra* genome using flow cytometry. Within this 992 Mb, nearly 94% of the single-copy orthologs prevalent in the Fabales clade were identified, highlighting the robustness of the assembly and the comprehensive genomic representation. In addition, despite the fragmented nature of the resulting information (~308 thousand scaffolds), the size of the obtained scaffolds (N50: 33,047 nt) enabled an efficient gene prediction, identifying 60,220 loci encoding high-confidence proteins. This value is higher than that found in the soybean genome (46,430 loci in 1.1 Gb [59]) and lower than that found in *A. hypogaea* peanuts (66,469 loci in ~2.7 Gb [60]), important legumes that are also tetraploid, like *S. scabra*.

A significant portion (approximately 60%) of the genome examined in this study is associated with regions containing transposable elements. The number of mobile elements in the plant clade varies greatly. Approximately 85% of the maize and barley genomes, for example, are composed of these entities [61,62,63], whereas in Arabidopsis, this proportion decreases to 20% [64]. The great portion of the *S. scabra* genome occupied by transposons may have a profound impact on its ecophysiological dynamics and evolutionary processes. The scientific literature suggests that environmental stresses—which are common in an environment as extreme as Caatinga—can activate the transposons’ action [65]. Thus, such elements can move to new genomic coordinates, which may cause changes in functional genes, resulting in positive or harmful evolutionary effects [66,67]. This is because the insertion of mobile elements within coding regions can inactivate them or produce an alternative splicing pattern, generating new proteins whose impact will be evolutionally evaluated [68]. From another perspective, insertion of transposons in regions close to genes may result in new control mechanisms, thus altering their expression [66]. Due to the Caatinga’s characteristic conditions of elevated temperatures, susceptibility to areas with increased salinity, and limited water availability (along with other edaphoclimatic factors that challenge plant physiology), the examination of the impact of mobile elements in *S. scabra* has emerged as a promising research area. This endeavor aligns with the newly initiated molecular journey that focuses on comprehending this species.

Regarding the mobile elements’ classification, the “LTR/Gypsy-type” and “Retrotransposons” were the most abundant annotated group. “LTRs” have a substantial presence in plant genomes, making up to 75% of the nuclear DNA [69]. As with all retrotransposons, this group replicates using an RNA intermediate (copy and paste mechanism). Following genomic integration of the newly generated copies, an extension of the host genome occurs. There is great availability of data showing that the mentioned process is among the main drivers of the evolution of genome size, resulting in large genomes—such as the genome of *S. scabra*—in species that are permissive of the LTRs’ accumulation [70].

### 4.2. Atlas of Resistance Proteins

Owing to their sessile lifestyle, plants are constantly exposed to biological threats caused by a wide range of pathogens. In the present study, approximately 2.6% of the *S. scabra* conceptual high-confidence proteome was associated with resistance proteins. These proteins, called “R” (“resistance”) and “PRR” (“pattern recognition receptors”), exert biological protection mechanisms using different strategies and cellular compartments [71]. Briefly, PRRs are proteins located in the plasma membrane; they act in the detection of PAMPs (patterns associated with pathogens or microorganisms) and trigger PTI (PAMP-triggered immunity). In this molecular warfare, pathogens use proteins called effectors, which prevent the PTI action, resulting in ETS (effector-triggered susceptibility). As a plant “countercoup”, if ETS is established, the effectors are likely to be recognized by intracellular immune receptors represented by “R” proteins—the second layer of plant defense—which are responsible for ETI (effector-triggered immunity) development.

The set of “R” and “PRR” proteins was diverse. Overall, 11 of the 13 possible groups were identified. Santana-Silva and Micheli [48] suggested that the quantitative and qualitative composition of these plant proteins are variant and species-specific. In legumes, the authors showed this by comparing the soybean and *Medicago truncatula* genomes. Our data, compared to those of Santana-Silva and Micheli [48], reinforce this species-specificity for legumes.

Interestingly, about 53% of the “R” and “PRR” proteins from *S. scabra* showed orthological relationships with soybean defense proteins. Due to the different ecological niches of these species, the phylogenetic distance, and the high evolutionary pressure that occurs during plant–pathogen interactions, it would be expected that such genes would have high diversity and variability among themselves. In a more specific context, we also observed that 19 orthologs between *S. scabra* and soybean were co-located—in silico—with soybean QTL regions associated with Asian soybean rust resistance. These elements become raw material for future studies that aim to develop more resistant *S. scabra* accessions to different biological threats, especially the fungus *Colletotrichum gloeosporioides*. In addition, it is worth noting that about half of these orthologs present a “many-to-one” orthology relationship in relation to soybean, suggesting that *S. scabra* has a more robust molecular defense “arsenal” compared to the other legumes mentioned above.

### 4.3. S. scabra as a Potential Source of Terpenes

Legumes act as reservoirs of secondary metabolites. These compounds can provide advantages for both the producing organisms and those that consume them (in the form of pharmaceuticals and nutraceuticals), thereby fostering health benefits. Due to the limited availability of studies analyzing the bromatology of *S. scabra* (despite it being used as forage), very little was known about its potential biochemical richness.

Our analyses revealed that the *S. scabra* genome anchors BGCs associated with terpene and saccharide synthesis, in addition to 11 BGCs whose chemical class has not been determined. In plants, there are few reports of these functional genomic structures compared with other clades [72]. Identification of this phenomenon of functional gene clustering has led to the development of new strategies driven by genomics that aim to discover metabolic pathways with biotechnological potential.

Because the term “saccharide” encompasses a large number of important compounds of primary metabolism, we will focus here on terpenes—a group of compounds of secondary metabolism with multifunctional action, although with more restricted functionality compared to sugars. According to Pichersky and Raguso [73], plants from different evolutionary lineages synthesize different terpenes, which are associated with defensive actions or the beneficial organisms’ attraction. Terpene diversity is related to a plant’s species, its biological “enemies” or mutualistic “friends”, and the biome in which the plant thrives [73]. Our analyses did not return data on which type of terpenes (monoterpenes, hemiterpenes, sesquiterpenes, etc.) are genomically encoded in the *S. scabra* genome. Nonetheless, it is plausible that they serve as pivotal components in the ecophysiology of the studied species, potentially displaying species-specific attributes. The biological significance of these terpenes, along with their potential biotechnological or commercial utility, requires comprehensive investigation. Notably, numerous Caatinga species have been extensively utilized in traditional medicine and the commercial production of herbal commodities.

### 4.4. Evaluation of Gene Families in Terms of Evolutionary Dynamics and Possible Ecophysiological Impacts

The 22,681 gene families encoded in the *S. scabra* genome could be categorized into at least two groups of biological importance: those that are specific to it (in relation to the pool of 12 species evaluated) and those that significantly expanded in relation to the quantity of its immediate ancestor shared with *A. hypogaea* (its closest phylogenetic relative used in the analysis). Such gene sets can be considered highly informative for deciphering key aspects of the legume that is the focus of the present study.

Regarding the first group, GO terms enrichment analyses were associated with important and informative biological processes, such as those related to terpene metabolism and cell wall dynamics. In relation to terpenes, this result reinforces the importance of BGCs identified in the *S. scabra* genome and associated with the synthesis of these compounds. In the previous subsection, the importance of terpenes in plant defense was mentioned. In addition, scientific reports showed their participation in the response to abiotic stresses, such as drought (a common condition in the Caatinga biome). According to Munné-Bosch et al. [74], diterpenes exert important antioxidant actions in plants under drought conditions, as observed for *Salvia officinalis*.

The cell wall can be considered a strategic structure for survival in extreme environments, such as the Caatinga, ensuring protection against harmful biological interactions and non-ideal edaphoclimatic conditions. The referenced polymeric structure can serve as a pre-formed structural barrier as well as an induced defense barrier. To succeed in infectious processes, pathogens need to bypass the cell wall and other preformed barriers to establish a pathogenic relationship with the host [75]. Functional characterization studies (obtaining transgenics or mutants) indicate that modification of the cell wall composition can result in resistance or susceptibility phenotypes in host plants (for a review, see Houston et al. [75]). In the context of abiotic stresses—mainly those that cause cell dehydration, such as drought and high salinity (common in the Caatinga environment)—the cell wall plays a fundamental role in acclimatization to these conditions. Many enzymes that promote complex modifications in this structure have been reported to be active under the mentioned situations (for a review, see [76,77]).

In another context, there was a significant expansion of 674 gene families in *S. scabra* relative to those of their last common ancestor with *A. hypogaea*. The biological processes enriched for this gene family pool provide evidence of the action of natural selection. Plants in the search for adaptation to unfavorable conditions notoriously use several of these biological strategies. Processes associated with reactive oxygen species (ROS) metabolism, for example, are widely studied. Oxidative stress is a complex chemical and physiological phenomenon considered a secondary aspect of practically all (a)biotic stresses in higher plants. This phenomenon results from the overproduction and accumulation of ROS, which causes severe damage to cell structures [78]. According to Hasanuzzaman et al. [79], plants suppress high ROS levels through endogenous mechanisms. They can be enzymatic (e.g., superoxide dismutase, catalase, peroxidases, etc.) or non-enzymatic (e.g., ascorbic acid, glutathione, non-amino acids, etc.). Data from our group on the transcriptomics of the 85/UNEB accession (the same as that studied in the present work) under irrigation suppression conditions indicated that it actively invested in the non-enzymatic mechanism for acclimatization to the imposed condition [13]. Maintaining an optimal ROS level in the cell allows for proper redox reactions and regulation of several essential processes such as plant growth and development [79].

Terms associated with the immune system (such as “immune system process” and “positive regulation of systemic acquired resistance”) were also enriched, considering the 674 significantly expanded gene families. In previous analyses (item “Resistance proteins atlas”), we reported that 2.6% of the conceptual high-confidence *S. scabra* proteome was associated with resistance proteins, which are involved in triggering PTI and ETI. In addition, in the pool of 674 gene families scrutinized here, there were proteins involved in other defense actions. This suggests the robustness of the 85/UNEB accession against possible pathogens, raising it to the level of a source of valuable genes for transfer to other economically important legumes.

Among other enriched biological processes, those associated with the biosynthesis of secondary compounds (e.g., “trehalose biosynthetic process”) or amino acids (e.g., “proline biosynthetic process”) are also worth mentioning. Regarding the first aspect, several studies have shown positive correlations between trehalose overproduction and better performance of the organisms analyzed under drought and high-salinity conditions [80,81], edaphoclimatic factors commonly found in the Caatinga biome. Considering the second category, the role of proline in plants is well known in the scientific literature. This amino acid plays a beneficial role in plants exposed to various stressful conditions. It acts as an important osmolyte (whose production is a key strategy in acclimatization to stresses caused by dehydration) and performs other functions such as a heavy metal chelator, antioxidant, and signaling molecule [82]. Many scientific reports have revealed that transgenic plants—particularly those that overexpress genes associated with proline biosynthesis—perform better when subjected to abiotic stresses [83]. Physiological data for the *S. scabra* 85/UNEB accession showed that its root system invested in proline synthesis for water deficit acclimation [13].

Finally, the dated phylogenetic tree and the comparative phylogenomic analysis (which indicated that 85% of the significantly expanded gene families in *S. scabra* were also significantly expanded in *A. hypogaea*) reinforced the close kinship between the *Stylosanthes* and *Arachis* genera. The tree was generated from the aggregation of individual estimates of each orthogroup identified by the software Orthofinder v2.5.5 [43]. This methodology is different and more current than its counterpart, which uses single-copy genes for kinship analyses between species. Several authors, such as Cardoso et al. [18,84,85], consider the genera *Stylosanthes* and *Arachis* as phylogenetic sister groups.

### 4.5. SscAQPs: Mining, Characterization, Transcriptomics, and Possible Impacts in S. scabra under Water Deficit

Aquaporins are a protein group of great importance. They form channels across cell membranes dedicated to transporting water and neutral solutes (represented by small molecules such as carbon dioxide, boron, silicon, etc.) [20]. SscAQPs genomic mining revealed that *S. scabra* contains four large groups of aquaporins (TIP, PIP, NIP, SIP). Compared to species of its sister genus *Arachis* (*A. duranensis* and *A. ipaensis* [51], in addition to *A. hypogaea* [52]), the pool of SscAQPs differed qualitatively due to the absence of XIP group members. In quantitative terms and compared to other tetraploid legumes, *S. scabra* had a lower number of canonical aquaporins (57) compared to soybean (75 aquaporins [86]) and *A. hypogaea* (64 aquaporins [52]). For all legumes mentioned above, TIP and PIP were the most abundant groups. This suggests a global pattern of abundance for these groups in the legume clade.

Regarding the SsAQPs’ gene structure, our data revealed that, within the group, the number and extension of exons varied little; in contrast, the intron range oscillated greatly. This is similar to what has been reported for *A. duranensis* and *A. ipaensis* [51]. The average number of introns (~2.7) in SscAQP genes was considerable, which could affect the generation of diversity in these proteins by the alternative splicing mechanism. Mapping of transcriptomic data on genomes revealed that the extent of alternative splicing in plants ranges from 42% to 61% (for a review, see Shang et al. [87]). Despite the “1:1” ratio between loci and isoforms encoding SscAQPs (i.e., 57 isoforms were associated with 57 loci) detected by the gene prediction pipeline, our transcriptomic data suggest that there is a greater diversity of aquaporin isoforms in the *S. scabra* genome. Considering only the SscAQPs upregulated under water deficit (24 h treatment), the 14 isoforms were associated with 11 loci. In the context of transcriptomics, the PIP group was the most upregulated in the *S. scabra* response to the 24 h treatment with water deficit. Regarding TIPs (the second group with the highest number of upregulated isoforms), PIP showed almost twice as many upregulated members in the evaluated RNA-Seq libraries (seven isoforms with their expression additionally validated by qPCR). Pharmacological studies have shown that PIPs may contribute up to 85% of root hydraulic conductivity [21]. Reverse genetic assays indicated that a single isoform of AQP in Arabidopsis, called AtPIP2;2, accounts for approximately 14% of the root hydraulic conductivity in this plant [88]. Thus, our data suggest that SscAQPs’ PIPs may play a crucial role in the acclimatization of *S. scabra* to water deficit conditions. This makes them potential targets for future functional characterization assays and biotechnological applications.

Finally, considering other structural aspects of SsAQPs, the two NPA motifs and the composition of the ar/R selectivity filters were analyzed. Such regions strongly affect the solvent/solute specificity and substrate transport through channels formed by aquaporins [89]. In contrast to the NPA motif conservation in all groups of searched SscAQPs, variation occurred in ar/R filters for both intra- and inter-groups. The exception was the SscAQP PIP group, in which all members showed hydrophilic amino acids F/H/T/R, confirming their involvement in water transport and emphasizing the importance of SscAQP PIPs. Ar/R filter with the constitution “F/H/T/R” is also found in AqpZ, a highly efficient water transport aquaporin identified in *E. coli* [90]. This was also observed in PIPs of other plants [51]. The variation found in the ar/R filters of the other groups of SscAQPs is associated with the multifaceted action of aquaporins, which can transport small gas molecules and other small neutral solutes.

## 5. Conclusions

In the present study, we successfully integrated omics approaches to shed light on some ecophysiological aspects of stress tolerance/resistance, as well as to probe the genomic landscape and potential metabolic wealth of *S. scabra*, an orphan plant found in the Caatinga biome. The recently inaugurated molecular journey of this plant shows that its genome is mostly (~59%) composed of transposable elements, a trait widely spread in the plant genomes analyzed so far. Regarding the protein-coding region (~38%), we identified 118,905 candidate loci, of which 60,220 were considered high-confidence loci. The categorization and study of information anchored by these structures allowed us to understand the important aspects of the ecophysiogical robustness of *S. scabra*. Gene families that are specific to the studied organism and those significantly expanded throughout its evolution participate in important mechanisms that ensure the adaptability of *S. scabra* to the extreme Caatinga environment. Furthermore, we observed a diverse defense protein set that positively affects the reported resistance of *S. scabra* to some pathogens. The analyzed genome also anchored BGCs associated with terpene biosynthesis, which may be species-specific. The biological, biotechnological, and commercial potentials of these genomic entities require thorough analysis. We also found that PIP-type SsAQPs—despite being genomically the second most abundant SsAQPs group—stood out as the most upregulated aquaporin group in the *S. scabra* response to water deficit. PIP-type SsAQPs have structural characteristics that are associated with water transport, aiding in *S. scabra* hydration when subjected to water deficit conditions. Finally, the genome assembly of this clade will provide valuable genomic resources for research, conservation, and breeding studies of *S. scabra,* the *Stylosanthes* genus, and other legume plants, thus benefiting both basic and applied plant biologists.

## Data Availability

The genomic and RNA-Seq raw reads substantiating the findings of this article are accessible in the NCBI Bioproject repository (https://www.ncbi.nlm.nih.gov/bioproject/), under the accession PRJNA924790, and in the NCBI SRA (https://www.ncbi.nlm.nih.gov/sra) repository, under the accession PRJNA837909, respectively.

## Supplementary Materials

The following supporting information can be downloaded at: https://www.mdpi.com/article/10.3390/plants12183246/s1, File S1: Melting curves for target transcripts analyzed in the present study; File S2: Evaluation of *Stylosanthes scabra* genome assembly and annotation completeness by BUSCO analysis; File S3: qPCR relative expression report by REST software; Figure S1: Some of the biological processes enriched for the set of gene families specific (in relation to the other analyzed species) to *Stylosanthes scabra*. Legend: Bubble color indicates the *p*-value (legend in the upper right-hand corner); “Log size” indicates the frequency of the GO term in the background data used (bubbles of more general terms are larger); Figure S2: Sequence alignment of *Stylosanthes scabra* aquaporins highlighting important and conserved structural regions; Table S1: High-confidence gene characterization using 11 structural parameters; Table S2: *Stylosanthes scabra* biosynthetic gene clusters characterization; Table S3: SscAQPs (*Stylosanthes scabra* aquaporins) gene characterization, presenting key structural elements composition, eleven common gene structural parameters, and subcellular location, isoelectric point (pI), and molecular weight (MW) for respective proteins; Table S4: Upregulated aquaporins in *Stylosanthes scabra* under water deficit conditions as determined by RNA-Seq analysis; Table S5: Reference genes and target aquaporins used in the qPCR relative expression analysis, presenting their respective transcript modulation, categorization, ID in genomic and transcriptomic platforms, primer pair sequences, RNA-Seq expression data, and primer pair efficiency values. Legend: UR (upregulated); PIP (plasma membrane intrinsic proteins).

## Abbreviations

GO terms (gene ontology terms); OGs (orthologous groups); PFAM (protein family); EC (Enzyme Commission); nT & nPCG (non-transposon and non-protein coding gene sequences); R-gene (resistance-gene); PRR gene (pattern recognition receptors); BGCs (biosynthetic gene clusters); SscAQPs (*Stylosanthes scabra* aquaporins); GO term (gene onthology term); TIPs (tonoplast intrinsic proteins); PIP (plasma membrane intrinsic proteins); NIP (nodulin 26-like intrinsic proteins); SIP (small basic intrinsic proteins); XIP (uncharacterized intrinsic proteins).
