# Supplementary material for: Bridging the Gap: Combining Genomics and Transcriptomics Approaches to Understand Stylosanthes scabra, an Orphan Legume from the Brazilian Caatinga
Source: Plants (Basel). 2023 Sep 13;12(18):3246. doi: 10.3390/plants12183246 (PMC10535828; doi:10.3390/plants12183246)
Supplement: Supplementary file 1 [file plants-12-03246-s001.zip › Supplementary Material 3.docx]

# Relative Expression Report

## Assay Parameters

| **Parameter** | **Value** |
| --- | --- |
| Iterations | 1000 |

## Results

| **Aquaporin ID** | **Aquaporin group** | **Type** | **Reaction Efficiency** | **Expression** | **Std. Error** | **95% C.I.** | **P(H1)** | **Result** |
| --- | --- | --- | --- | --- | --- | --- | --- | --- |
| Ssc_29553\|c0_g1_i5 | PIP | TRG | 0,9104 | 2,708 | 0,881 - 12,553 | 0,517 - 16,973 | 0,019 | UP |
| Ssc_65911\|c1_g1_i3 | PIP | TRG | 0,9490 | 3,759 | 0,761 - 34,065 | 0,487 - 97,031 | 0,048 | UP |
| Ssc_29553\|c0_g1_i7 | PIP | TRG | 0,9412 | 2,472 | 0,642 - 7,688 | 0,337 - 23,715 | 0,047 | UP |
| Ssc_67211\|c1_g1_i3 | PIP | TRG | 0,9585 | 2,109 | 0,793 - 4,854 | 0,558 - 11,351 | 0,035 | UP |
| Ssc_29553\|c0_g1_i9 | PIP | TRG | 0,9412 | 1,342 | 0,972 - 1,961 | 0,693 - 2,395 | 0,023 | UP |
| Ssc_65911\|c1_g1_i5 | PIP | TRG | 0,9182 | 1,705 | 1,245 - 2,205 | 0,953 - 3,683 | 0,001 | UP |
| Ssc_29553\|c0_g2_i3 | PIP | TRG | 1,0 | 1,353 | 0,949 - 1,887 | 0,747 - 2,888 | 0,024 | UP |
| Ssc_β-tubulin | - | REF | 1,1 | 1,116 | - | - | - | - |
| Ssc_Ubiquitin | - | REF | 1,0 | 0,896 | - | - | - | - |

**Legend:**

P(H1) – Probability of alternate hypothesis that difference between sample and control groups is due only to chance.
TRG – Target gene
REF – Reference gene

PIP – Plasma membrane intrinsic proteins

## Interpretation

|  |
| --- |
|  |
| **Ssc_29553\|c0_g1_i5** is UP-regulated in sample group (in comparison to control group) by a mean factor of 2,708 (S.E. range is 0,881 - 12,553). |
| **Ssc_29553\|c0_g1_i5** sample group is different to control group. P(H1)=0,019 |
|  |
| **Ssc_65911\|c1_g1_i3** is UP-regulated in sample group (in comparison to control group) by a mean factor of 3,759 (S.E. range is 0,761 - 34,065). |
| **Ssc_65911\|c1_g1_i3**sample group is different to control group. P(H1)=0,048 |

**Ssc_29553|c0_g1_i7** is UP-regulated in sample group (in comparison to control group) by a mean factor of 2,472 (S.E. range is 0,642 - 7,688).

**Ssc_29553|c0_g1_i7** sample group is different to control group. P(H1)=0,047

**Ssc_67211|c1_g1_i3** is UP-regulated in sample group (in comparison to control group) by a mean factor of 2,109 (S.E. range is 0,793 - 4,854).

**Ssc_67211|c1_g1_i3** sample group is different to control group. P(H1)=0,035

**Ssc_29553|c0_g1_i9** is UP-regulated in sample group (in comparison to control group) by a mean factor of 1,342 (S.E. range is 0,972 - 1,961).

**Ssc_29553|c0_g1_i9** sample group is different to control group. P(H1)=0,023

**Ssc_65911|c1_g1_i5** is UP-regulated in sample group (in comparison to control group) by a mean factor of 1,705 (S.E. range is 1,245 - 2,205).

**Ssc_65911|c1_g1_i5** sample group is different to control group. P(H1)=0,001

**Ssc_29553|c0_g2_i3** is UP-regulated in sample group (in comparison to control group) by a mean factor of 1,353 (S.E. range is 0,949 - 1,887).

**Ssc_29553|c0_g2_i3** sample group is different to control group. P(H1)=0,024
