# Supplementary figures and images for: Bridging the Gap: Combining Genomics and Transcriptomics Approaches to Understand Stylosanthes scabra, an Orphan Legume from the Brazilian Caatinga
Source: Plants (Basel). 2023 Sep 13;12(18):3246. doi: 10.3390/plants12183246 (PMC10535828; doi:10.3390/plants12183246)

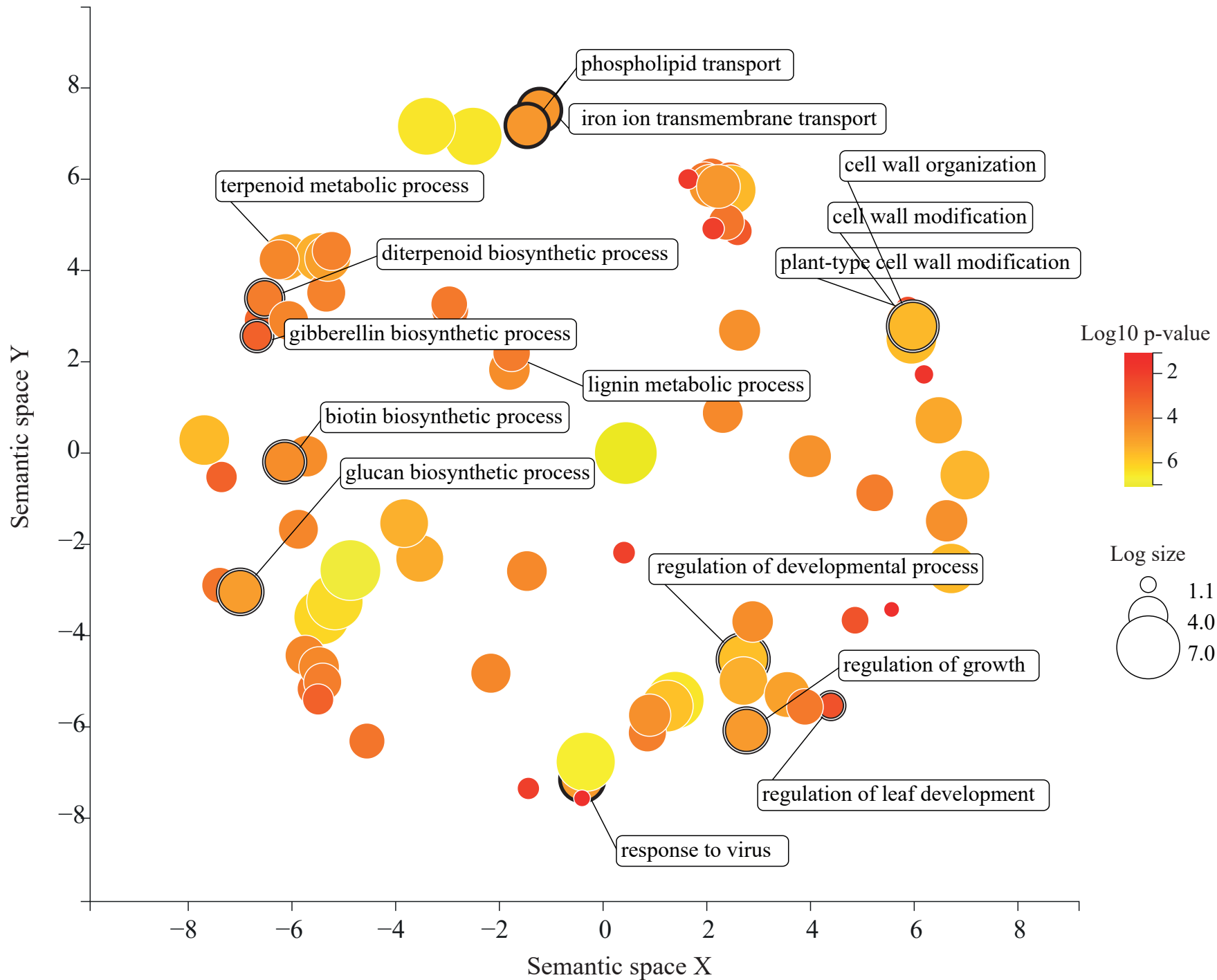

Supplement: Supplementary file 1 [file plants-12-03246-s001.zip › Supplementary Figure S1.pdf]

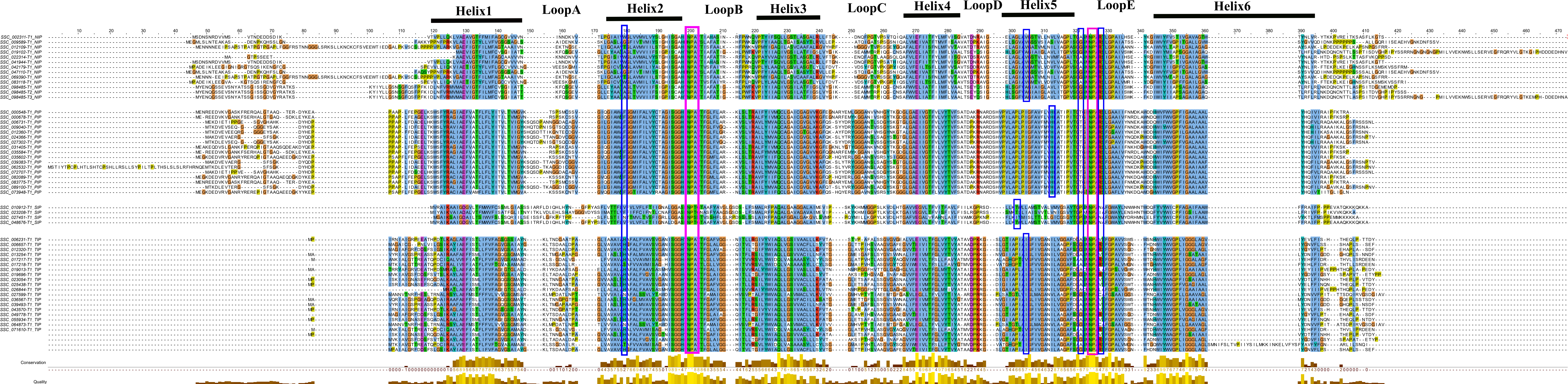

Supplement: Supplementary file 1 [file plants-12-03246-s001.zip › Supplementary Figure S2.pdf]

Ssc_29553|c0_g2_i3
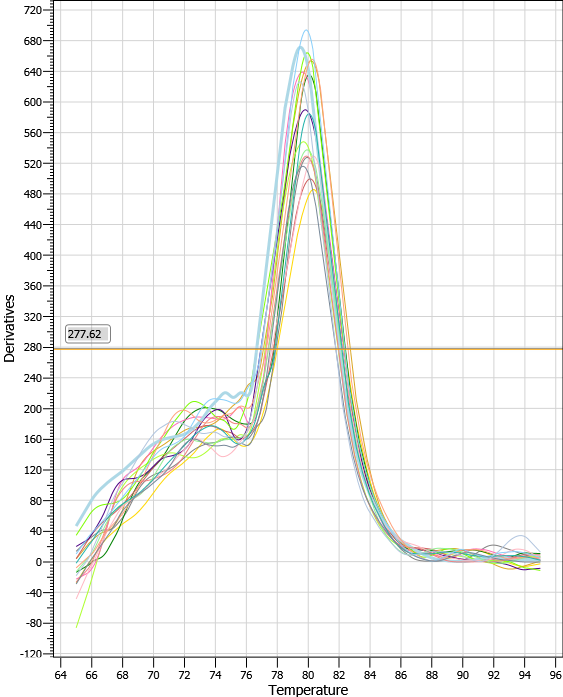


Ssc_29553|c0_g1_i5
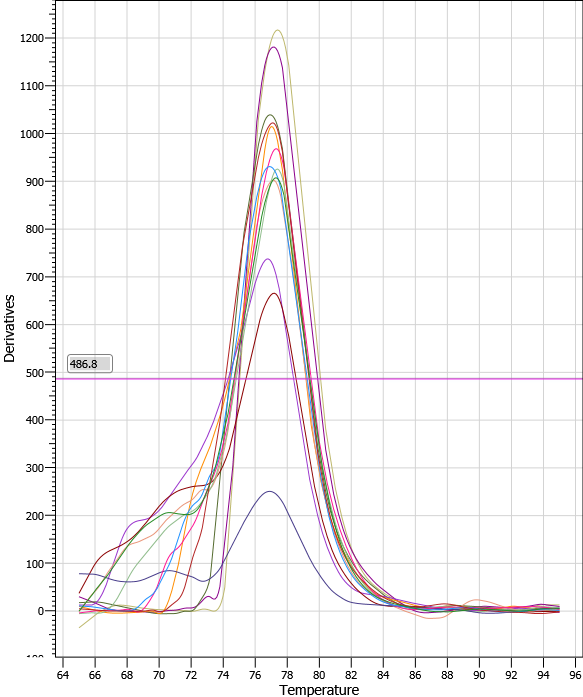


Ssc_29553|c0_g1_i7
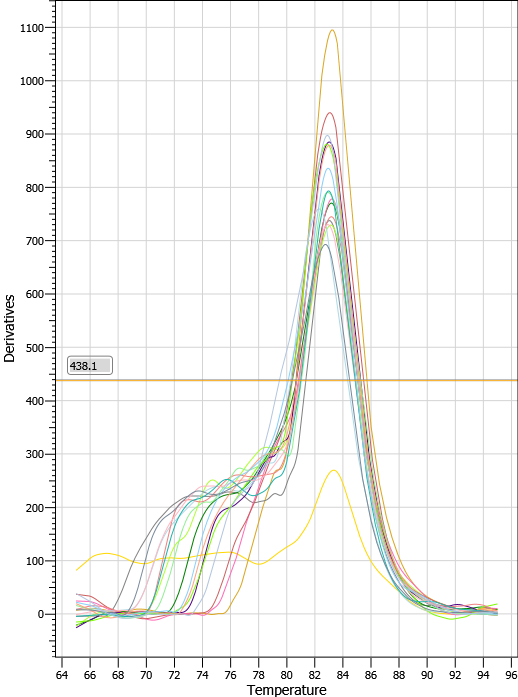


Ssc_29553|c0_g1_i9
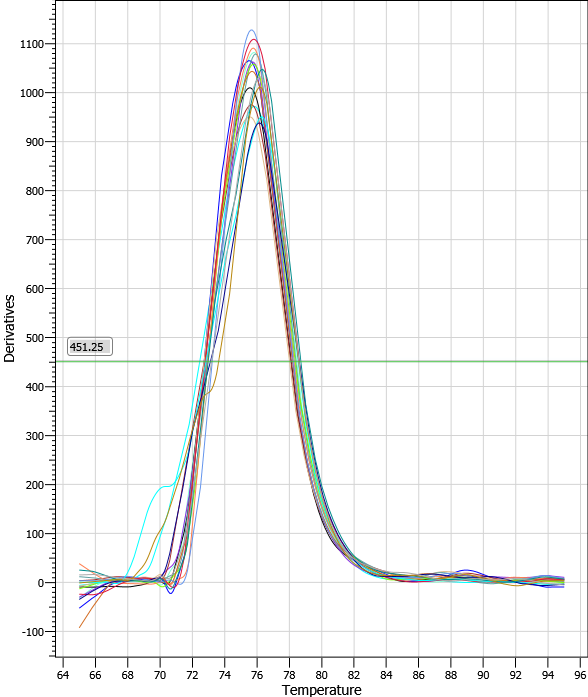


Ssc_65911|c1_g1_i5
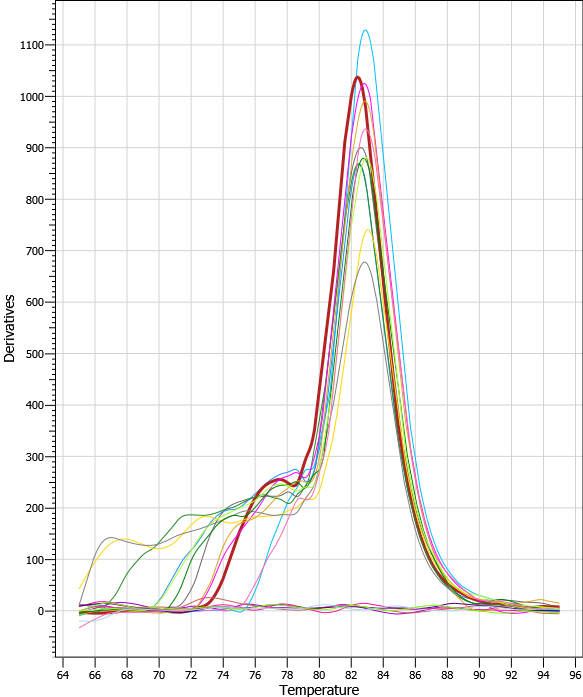


Ssc_65911|c1_g1_i3
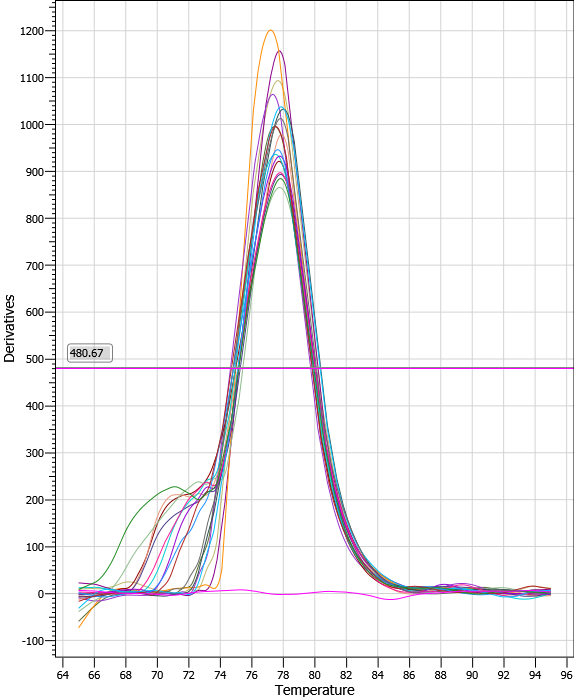


Ssc_67211|c1_g1_i3
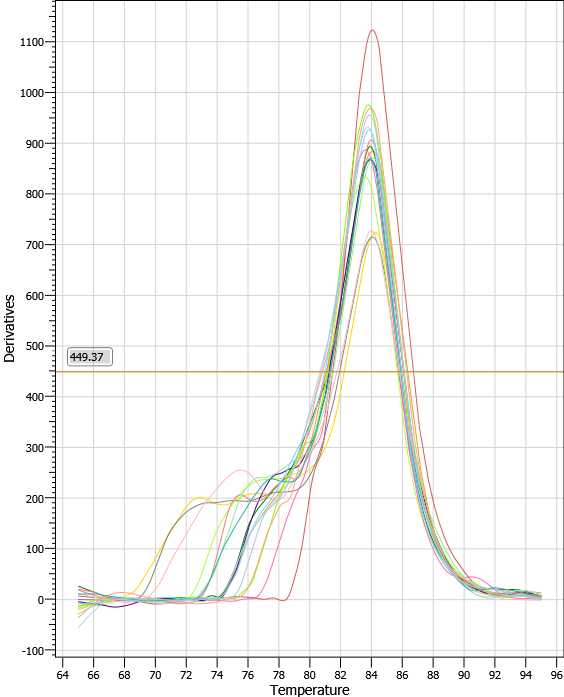

Supplement: Supplementary file 1 [file plants-12-03246-s001.zip › Supplementary Material 1.docx]
