# Supplementary material for: Bridging the Gap: Combining Genomics and Transcriptomics Approaches to Understand Stylosanthes scabra, an Orphan Legume from the Brazilian Caatinga
Source: Plants (Basel). 2023 Sep 13;12(18):3246. doi: 10.3390/plants12183246 (PMC10535828; doi:10.3390/plants12183246)
Supplement: Supplementary file 1 [file plants-12-03246-s001.zip › Supplementary Material 2.doc]

| SHORT SUMMARY OF THE PROJECT | |
| --- | --- |
| **Job ID** | 202204122142-79HT3R22YV33F4JV |
| **Project name** | *Stylosanthes scabra* genome assembly |
| **Selected program** | BUSCO_v5 |
| **Selected ortholog set** | Fabales |

|         COMPLETENESS ASSESSMENT RESULTS | |
| --- | --- |
| **Total number of core genes queried** | 5366 |
| **Number of core genes detected** | |
| **Complete** | 4973 (92.68%) |
| **Complete + Partial** | 5068 (94.45%) |
| **Number of missing core genes** | 298 (5.55%) |
| **Average number of orthologs per core gene** | 1.89 |
| **% of detected core genes that have more than 1 ortholog** | 74.96 |
| **Scores in BUSCO format** | C: 92.7% [S: 23.2%, D: 69.5%]; F: 1.8%; M: 5.5% |
